# A new method for evaluating air quality using an ideal grey close function cluster correlation analysis method

**DOI:** 10.1038/s41598-021-02880-1

**Published:** 2021-12-02

**Authors:** Xiaoling Ren, Zhenfu Luo, Shuyu Qin, Xinqian Shu, Yuanyuan Zhang

**Affiliations:** 1 China University of Mining and Technology-Beijing, Beijing, 100083 China; 2 School of Chemical Engineering and Technology, China University of Mining and Technology, Xuzhou, 221116 Jiangsu China; 3 Yinchuan College, China University of Mining and Technology, Yinchuan, 750411 Ningxia China

**Keywords:** Environmental impact, Atmospheric chemistry

## Abstract

To scientifically and reasonably evaluate air quality with a large amount of monitored data, this paper proposes a new evaluation method called ideal grey close function cluster correlation analysis (*IGCFCCA*). Taking the air quality in Ningxia Province, China, as an example, according to China’s air quality standard, SO_2_, NO_2_, PM_10_, PM_2.5_ and O_3_ are selected as evaluation indexes to perform the evaluation. The results show that the air quality in this region in 2018 can be divided into three classifications, among which the relatively poor air quality in March, April and May is the first classification, the better air quality in August and September is the third classification, and the air quality in other months falls under the second classification. Correlation analysis is used to qualitatively determine that these three classifications correspond to first-level air quality in China’s air quality standard, and the correlation degree, which is the distance between the three classifications and the first-level air quality, is quantitatively determined. Specifically, the correlation degrees of the first-classification, second-classification and third-classification of air quality are 0.674, 0.697 and 0.71, respectively. The research results indicate potential directions and objectives for air quality management to achieve scientific management.

## Introduction

The air environment is a dynamic and complex system. The air quality is influenced by some pollutants, such as SO_2_, NO_2_, PM_10_, and O_3_. The concentrations of these pollutants are changing constantly. However, the monitored data used in analyses are usually collected in a certain period, and examples include one-hour average, few-hour average, one-month average and one-year average data. Instantaneous data collected every minute or second are difficult to collect and analyse. Therefore, this collection approach is considered a grey system. In a grey system, some information is known, and some information is unknown^1–7^.

At present, China’s air quality standard (*GB3095-2012*) divides air quality into two levels and stipulates the concentrations of pollutants in first-level and second-level air^8,9^. The concentrations of pollutants are comparatively lower in first-level air, and they are higher in second-level air. The major pollutants include SO_2_, NO_2_, PM_10_, O_3_, and others. However, when people evaluate air quality according to *GB3095-2012*, there may be some problems. First, according to the national standard, the common evaluation methods can only determine which level the current air is associated with. However, there is no analysis of how much the current air belongs to the level, and it is not clear how far the current air is from the standard level. The space for improving the current air quality is also very vague. It is necessary to develop a method to quantitatively calculate the correlation degree, which is the distance between the current air and the two levels of air standards. Second, to determine the air quality in a certain area in a period of time, the concentrations of pollutants are usually monitored every day. However, the amount of monitored data is very large. Obviously, if people compare and analyse each recorded value, the workload will be very large, and tasks will be almost impossible to complete. Therefore, people usually calculate the average value of the data first and then analyse the average. However, among so many monitored data, which data should be taken as a group for average calculation is a problem. In other words, determining how to scientifically classify data is the key. Data with similar characteristics can be classified into one group. These different classifications can be analysed and evaluated. Therefore, the results of the analysis can be scientific.

At present, there are many methods for comprehensively evaluating atmospheric environmental quality, including the air pollution index (API) method, ambient air quality index (AQI) method, single factor index method, green air pollution comprehensive index method, analytic hierarchy process, artificial neural network models, and fuzzy comprehensive evaluation method^8^. Due to the different evaluation principles of various evaluation methods, each method has unique advantages and disadvantages. Among them, the API and AQI methods are simple, intuitive and convenient to use but only applicable for evaluating the short-term air quality in cities^9^. The single factor index method is clear and easy to implement, but it cannot consider the air quality status as a whole, and the evaluation results are one dimensional^9^. Green's comprehensive air pollution index method is easy to understand and implement, but it is only applicable to areas where coal pollution is the main pollution type^9^. The analytical hierarchy process (AHP) is simple, practical and systematic, but quantitative results are limited; additionally, when there are many indicators, the statistics will be complex, and weights will be difficult to determine^9^. The artificial neural network evaluation method has the advantages of a fast operation speed, self-adaptation and strong fault tolerance, but the disadvantage is that when the data are poorly correlated, the evaluation results will exhibit homogenization phenomena^10–13^. IGCFCCA is a kind of fuzzy comprehensive evaluation method based on fuzzy mathematics, the fuzzy principle and the grey close function. The method can solve the common incomplete data problem and mainly assesses the relationships between uncertainty and incomplete information analysis, model building and forecasting. The method only needs a small amount of data and can achieve good prediction results.

In this paper, the IGCFCCA method is used to evaluate the air quality in Ningxia Province. The method can not only scientifically classify a large amount of data but also calculate the correlation degree between each classification and the relevant standard. This approach can provide an important basis for comprehensive environmental management. Moreover, this new method provides a scientific reference and an important basis for the establishment and optimization of other industry standards in the future.

## Basic principle and methods

A sample, which comes from the monitored data reports of some environmental management departments, is first classified by ideal grey close function cluster analysis. Then, the level of the sample is determined by grey correlation analysis, and comprehensive evaluation conclusions are established according to the correlation degree between the classification of the sample and the levels specified in *GB3095-2012*.

### The classification of the sample to be evaluated

#### Establishing the evaluation index sequence matrix for the selected sample

Let *S* be a sequence of clustering objects, i.e., *S* = {*s*_1_, *s*_2_…, *s*_*m*_}; *X* is a sequence of air-influencing variables, i.e., *X* = {*x*_1_, *x*_2_…, *x*_*n*_}; *x*_*ik*_ is the original monitoring data for *s*_*i*_ (*i* = 1, 2…, *m*) and *x*_*k*_ (*k* = 1, 2…, *n*); *i* and *m* represent the number of objects considered in clustering; *k* and *n* are the number of the influencing indexes which are the pollutants mentioned above. Accordingly, the following matrix can be established (Eq. 1).

1 $$ S = \begin{array}{*{20}c} {s_{1} } \\ {s_{2} } \\ \ldots \\ {s_{m} } \\ \end{array} \left[ {\begin{array}{*{20}c} {x_{11} } & {x_{12} } & \ldots & {x_{1n} } \\ {x_{21} } & {x_{22} } & \ldots & {x_{2n} } \\ \ldots & \ldots & \ldots & \ldots \\ {x_{m1} } & {x_{m2} } & \ldots & {x_{mn} } \\ \end{array} } \right] $$

#### Establishing the matrix of ideal-value grey close function clusters

Let *X*_0_ = {*x*_01_, *x*_02_…, *x*_0*n*_} be the ideal-value sequence corresponding to each influential index. The principle for determining the ideal value is as follows (Eqs. 2, 3, 4).

The first situation: The larger the influencing index (*x*_*k*_) is, the better the air quality is; in this case, the ideal value

2 $$ x_{0k} = \max \left\{ {x_{ik} ,i = 1,2, \ldots ,m} \right\},k = 1,2, \ldots ,n. $$

The second situation: The smaller the influencing index (*x*_*k*_) is, the better the air quality is; in this case, the ideal-value

3 $$ x_{0k} = \min \left\{ {x_{ik} ,i = 1,2, \ldots ,m} \right\},k = 1,2, \ldots ,n. $$

Third, the air quality is best when the influencing index (*x*_*k*_) displays a moderate value, and the ideal value is

4 $$ x_{0k} = {\text{M}}. $$

According to the ideal value *x*_0*k*_ (Eqs. 2, 3 or Eq. 4) and the original monitored data (*x*_*ik*_), the grey close function value *y*_*ik*_ is calculated by using (Eq. 5).

5 $$ y_{ik} = \frac{{x_{ok} }}{{x_{ik} }}\;\left( {i = 1,2, \ldots ,m;k = 1,2, \ldots ,n} \right) $$

where *x*_*ik*_ is the original monitored data and *x*_0*k*_ is the ideal value corresponding to the k-th influential index. Moreover, the function value *y*_*ik*_ is dimensionless, and *y*_*ik*_ ∈ [0,1]. *y*_*ik*_ denotes the correlation degree of *s*_*i*_ and *s*_0_ for the k-th index. Specifically, the larger *y*_*ik*_ is, the closer *s*_*i*_ is to the ideal value *s*_0_, and the smaller *y*_*ik*_ is, the farther *s*_*i*_ is from *s*_0_.

Thus, the following grey close matrix *Y* can be established (Eq. 6).

6 $$ Y = \left[ {\begin{array}{*{20}c} {y_{11} } & {y_{12} } & \ldots & {y_{1n} } \\ {y_{21} } & {y_{22} } & \ldots & {y_{2n} } \\ \begin{gathered} \ldots \hfill \\ y_{m1} \hfill \\ \end{gathered} & \begin{gathered} \ldots \hfill \\ y_{m2} \hfill \\ \end{gathered} & \begin{gathered} \ldots \hfill \\ \ldots \hfill \\ \end{gathered} & \begin{gathered} \ldots \hfill \\ y_{mn} \hfill \\ \end{gathered} \\ {y_{01} } & {y_{02} } & {...} & {y_{0n} } \\ \end{array} } \right] $$

In this case, *Y* is the grey close function value. Moreover, (*y*_01_, *y*_02_…, *y*_0*n*_) = (1,1…,1)_1×*n*_ is the ideal sequence, and the bigger *y*_*ik*_ is, the better *s*_*i*_ is; the biggest *y*_*ik*_ is equal to 1.

#### The classification of the sample to be evaluated

Because the influence of each influencing index is different, the weight of each influencing index needs to be considered. Let *P*_*i*_ be the comprehensive analysis value of *s*_*i*_. *P*_*i*_ can be expressed as follows (Eq. 7)

7 $$ P_{i} = \sum\limits_{k = 1}^{n} {Wy_{ik} } \left( {i = 1,2 \ldots ,m} \right) $$

where *W* is the weight of each influencing index, and since the number of indexes is *k*, the number of *W* values is also* k* (*W*_1_, *W*_2_…, *W*_*k*_). Corresponding, the following equation can be established (Eq. 8).

8 $$ W_{k} = \frac{{\sum\limits_{i = 1}^{m} {X_{{i{\text{k}}}} } }}{{\sum\limits_{i = 1}^{m} {\sum\limits_{k = 1}^{n} {X_{ik} } } }}\;\left( {k = 1,2 \ldots ,n} \right) $$

Based on the actual comprehensive analysis value *P*_*i*_, *P*_*j*_ = (*P*_1_, *P*_2_…, *P*_*m*_)^T^. The following equation (Eq. 9) can be used to calculate the grey close value *P*_*ij*_ of *P*_*i*_ in relation to *P*_*j*_.

9 $$ P_{ij} = \frac{{\min (p_{i} ,p_{j} )}}{{\max (p_{i} ,p_{j} )}}\;\left( {i,j = 1,2 \ldots ,m} \right) $$

Then,

10 $$ P = \left( {P_{ij} } \right)_{m \times m} . $$

If *P* (Eq. 10) satisfies the following three conditions: (1) reflexivity, where *P*_*ij*_ = 1 (*i* = *j*); (2) symmetry, where *P*_*ij*_ = *P*_*ji*_; and (3) normativity, where *P*_*ij*_ ∈ [0,1], we can select the appropriate threshold value from the *P* matrix, intercept the branches with weight values less than λ, which is the similarity coefficient^4,5^, and establish the classification $S_{t}^{\prime }$ (*t* = 1, 2…, *c*) when λ level meets the relevant requirement. $S_{t}^{\prime }$ represents each classification of the air in a given region. The following equations (Eqs. 11, 12) can be established.

11 $$ S_{t}^{\prime } = \left( {S_{1}^{\prime } ,S_{2}^{\prime } \ldots ,S_{c}^{\prime } } \right)^{{\text{T}}} $$

12 $$ S_{tk}^{\prime } = \left( {S_{t1}^{\prime } ,S_{t2}^{\prime } \ldots ,S_{tn}^{\prime } } \right) $$

where $S_{t}^{\prime }$ is the t-th classification, $S_{tk}^{\prime }$ is the kth index of the t-th classification, *t* is the number of classifications, and *k* is the number of influencing indexes.

$S_{tk}^{\prime }$ can be expressed in the following matrix form (Eq. 13).

13 $$ S_{tk}^{\prime } = \left[ {\begin{array}{*{20}c} {s_{11}^{\prime } } & {s_{12}^{\prime } } & \ldots & {s_{1n}^{\prime } } \\ {s_{21}^{\prime } } & {s_{22}^{\prime } } & \ldots & {s_{2n}^{\prime } } \\ \ldots & \ldots & \ldots & \ldots \\ {s_{cc}^{\prime } } & {s_{c2}^{\prime } } & \ldots & {s_{cn}^{\prime } } \\ \end{array} } \right] $$

### Correlation degree analysis of the sample to be evaluated

Let $S_{t}^{\prime }$ be the sample to be evaluated, and let *X* = (*x*_1_, *x*_2_…, *x*_*n*_), which is the influencing index set mentioned above and is the evaluation index used for $S_{t}^{\prime }$. Let ${\text{S}}_{0}^{\prime }$ be the stated air quality classification in the *GB3095-2012*. Then, the equation for the correlation coefficient is as follows (Eq. 14)^14^.

14 $$ \zeta_{t} (k) = \frac{{\mathop {\min }\limits_{t \in c} \mathop {\min }\limits_{k \in n} \left| {S_{t}^{\prime } (k) - {\text{S}}_{0}^{\prime } (k)} \right| + \epsilon \mathop {\max }\limits_{t \in c} \mathop {\max }\limits_{k \in n} \left| {S_{t}^{\prime } (k) - {\text{S}}_{0}^{\prime } (k)} \right|}}{{\left| {S_{t}^{\prime } (k) - {\text{S}}_{0}^{\prime } (k)} \right| + \epsilon \mathop {\max }\limits_{t \in c} \mathop {\max }\limits_{k \in n} \left| {S_{t}^{\prime } (k) - {\text{S}}_{0}^{\prime } (k)} \right|}} $$

where *ζ*_*t*_ (*k*) is the correlation coefficient and ε is the resolution coefficient, with a general value of 0.5^4,5^.

Moreover, the correlation degree (*R*_*t*_) equation is as follows (Eq. 15).

15 $$ R_{t} = \frac{1}{n}\sum\limits_{k = 1}^{n} {\zeta_{t} } (k) $$

The value of *R*_*t*_ is calculated by using (Eq. 15). The maximum value of *R*_*t*_ indicates that the sample to be evaluated has the highest correlation degree with the considered air quality level. Therefore, the sample is classified correspondingly.

## Air quality assessment—taking Ningxia Province in China as an example

### The classification of the samples to be evaluated

Monthly reports of the air quality in Ningxia Province in 2018 were provided by the Department of Ecology and Environment of Ningxia Province. The monthly report data were used to establish the cluster of samples *S* (Table 1) (Eq. 1). Each sample included five kinds of pollutants. Moreover, the concentrations of SO_2_, NO_2_, PM_10_ and PM_2.5_ were based on monthly averages calculated from 24-h averages, and the concentration of O_3_ was the monthly average calculated from the 8-h average values.

**Table 1:** Air quality in Ningxia Province in 2018.

Index	Monthly average concentrations of major monitored pollutants (μg/m^3^)				
	SO_2_ (*x*_1_)	NO_2_ (*x*_2_)	PM_10_ (*x*_3_)	PM_2.5_ (*x*_4_)	O_3_ (*x*_5_)
January			93	46	
February	40	27	86	40	104
March	30	32	167	55	129
April	18	27	159	47	141
May	14	23	150	45	162
June	14	24	74	26	178
July	9	17	81	29	160
August	10	20	56	25	150
September	13	27	65	26	129
October	19	37	87	39	112
November	27	43	155	57	83
December	32	37	141	50	76

*x*_1_ is the SO_2_ concentration; *x*_2_ is the NO_2_ concentration; *x*_3_ is the PM_10_ concentration; *x*_4_ is the PM_2.5_ concentration; and *x*_5_ is the O_3_ concentration. For these pollutants, the lower the concentration is, the better the air quality is.

As shown in Table 1, because the management department only provided some monitored data and the data in January are incomplete, only the data that are listed in the table from February to December can be effectively analysed. However, the focus of this study is on the new analysis and evaluation method (*IGCFCCA*), and almost all of the data can be analysed by this method.

According to (Eq. 3), the five ideal values are as follows: *x*_01_ is 9, *x*_02_ is 17, *x*_03_ is 56, *x*_04_ is 25, and *x*_05_ is 76. Based on the sample data in Table 1, the ideal-value grey close matrix (Eq. 6) can be obtained from (Eq. 5); according to (Eq. 8), the weights of *x*_1_, *x*_2_, *x*_3_, *x*_4_ and *x*_5_ are *w*_1_ = 0.06, *w*_2_ = 0.09, *w*_3_ = 0.34, *w*_4_ = 0.12, and *w*_5_ = 0.39, respectively. Consequently, the comprehensive analysis value *P*_*i*_ (*i* = 1, 2…, 11) (Table 2) of *S*_*i*_ is calculated with (Eq. 7). The grey close function value *y*_*ik*_ (Eq. 5) and the comprehensive analysis value *P*_*i*_ are shown in Table 2.

**Table 2:** Grey close function value and the comprehensive analysis value.

Index	*X*_1_	*X*_2_	*X*_3_	*X*_4_	*X*_5_	Comprehensive analysis value (*P*_*i*_)
*S*_1_	0.225	0.630	0.651	0.625	0.731	0.651
*S*_2_	0.300	0.531	0.335	0.455	0.589	0.464
*S*_3_	0.500	0.630	0.352	0.532	0.539	0.481
*S*_4_	0.643	0.739	0.373	0.556	0.469	0.482
*S*_5_	0.643	0.708	0.757	0.962	0.427	0.641
*S*_6_	1.000	1.000	0.691	0.862	0.475	0.673
*S*_7_	0.900	0.850	1.000	1.000	0.507	0.787
*S*_8_	0.692	0.630	0.862	0.962	0.589	0.736
*S*_9_	0.474	0.459	0.644	0.641	0.679	0.631
*S*_10_	0.333	0.395	0.361	0.439	0.916	0.590
*S*_11_	0.281	0.459	0.397	0.500	1.000	0.645

With *P*_*i*_ (*P*_1_, *P*_2_… and *P*_11_) as known numbers, *P*_*ij*_ (*j* = 1, 2…, 11) can be calculated from (Eq. 9). The corresponding elements of the grey similar matrix (Eq. 10) are shown in Table 3.

**Table 3:** Grey close values *P*_*ij*_.

*S*	*S*_1_	*S*_2_	*S*_3_	*S*_4_	*S*_5_	*S*_6_	*S*_7_	*S*_8_	*S*_9_	*S*_10_	*S*_11_
*S*_1_	1.0000										
*S*_2_	0.7127	1.0000									
*S*_3_	0.7389	0.9647	1.0000								
*S*_4_	0.7404	0.9627	0.9979	1.0000							
*S*_5_	0.9846	0.7239	0.7504	0.7520	1.0000						
*S*_6_	0.9673	0.6895	0.7147	0.7162	0.9525	1.0000					
*S*_7_	0.8272	0.5896	0.6112	0.6125	0.8145	0.8551	1.0000				
*S*_8_	0.8845	0.6304	0.6535	0.6549	0.8709	0.9144	0.9352	1.0000			
*S*_9_	0.9693	0.7353	0.7623	0.7639	0.9844	0.9376	0.8018	0.8573	1.0000		
*S*_10_	0.9063	0.7864	0.8153	0.8169	0.9204	0.8767	0.7497	0.8016	0.9350	1.0000	
*S*_11_	0.9908	0.7194	0.7457	0.7473	0.9938	0.9584	0.8196	0.8764	0.9783	0.9147	1.0000

The following information can be obtained from Table 3. If λ = 0.9^4,5^, *S*_2_, *S*_3_ and *S*_4_ correspond to the first classification $S_{1}^{\prime }$; *S*_7_ and *S*_8_ correspond to the third classification $S_{3}^{\prime }$; and the other *S* values correspond to the second classification $S_{2}^{\prime }$. *S*_2_, *S*_3_ and *S*_4_ are the samples for March, April and May, respectively, and *S*_7_ and *S*_8_ are the samples for August and September, respectively. Cluster $S_{tk}^{\prime }$ (Eq. 13) (Table 4) includes $S_{1}^{\prime }$, $S_{2}^{\prime }$ and $S_{3}^{\prime }$.

**Table 4:** The classifications of air.

Index	*x*_1_	*x*_2_	*x*_3_	*x*_4_	*x*_5_
First classification	20.67	27.33	158.67	49.00	144.00
Second classification	23.50	30.83	104.00	40.17	118.83
Third classification	11.50	23.50	60.50	25.50	139.50

The samples (Table 1) can be divided into three classifications, and the class-based approach provides two main advantages. First, if the data in each month are compared and analysed with the air standards, the workload will be large, and errors will easily accumulate. In contrast, only analysing the three classifications can greatly improve the work efficiency. Second, this classification method can be used to establish national or local standards. For example, actual statistical data over many years can be classified by this method, and the classification results can be used as new comparison standards, which would be beneficial to the analysis and evaluation of statistical data in the future.

### Sample evaluation and correlation degree analysis

**Figure 1:**
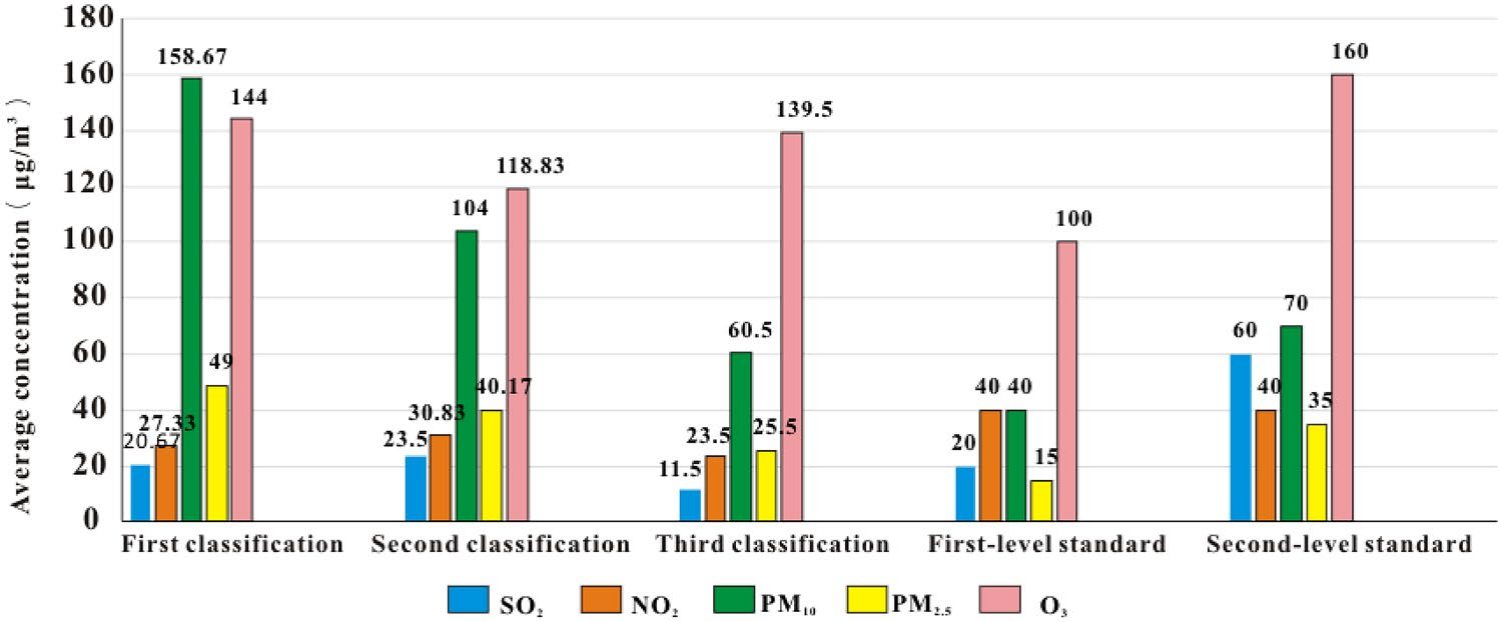
Comparison of the samples to be evaluated with the two levels of air standards.

In the former parts of the paper, the samples from each month in 2018 are divided into three classifications ($S_{1}^{\prime }$, $S_{2}^{\prime }$ and $S_{3}^{\prime }$). The concentrations of these pollutants in the air quality standard (*GB3095-2012*) are used for comparison, and the comparison of the data is shown in Fig. 1.

As shown in Fig. 1, compared with that in the first-level air standard, the SO_2_ concentration in the third-classification air standard is lower, and the NO_2_ concentrations in the three air classes are all lower than the concentration in the first-level air standard. In other words, the concentration of NO_2_ in the region meets the first-level air standard throughout the year, and the concentration of SO_2_ in August and September meets the first-level air standard. Therefore, according to the first-level air standard, the region should strengthen the management of PM_10_, PM_2.5_ and O_3_ emissions throughout the year, and the management of SO_2_ emissions in months other than August and September should be strengthened.

Compared with the second-level air, the concentrations of SO_2_, NO_2_, O_3_ in the three air classes are all lower than that in the second-level standard, the concentrations of PM_10_ and PM_2.5_ in the third classification of air are lower those in the second-level standard. In other words, the concentrations of NO_2_, SO_2_ and O_3_ in the region meet the second-level air standard throughout the year. Moreover, the concentrations of PM_10_ and PM_2.5_ in August and September meet the second-level air standard. Therefore, according to the second-level air standard, the region should strengthen the management of PM_10_ and PM_2.5_ emissions.

According to grey theory, the cluster data and the data (${\text{S}}_{01}^{\prime }$and ${\text{S}}_{02}^{\prime }$ from air quality standard) used for comparison must be initialized^4,5^, and the initial values are shown in Table 5.

**Table 5:** Data initialization results.

Index	*x*_1_	*x*_2_	*x*_3_	*x*_4_	*x*_5_
$S_{1}^{\prime }$	1.000	1.322	7.676	2.371	6.967
$S_{2}^{\prime }$	1.000	1.312	4.426	1.709	5.057
$S_{3}^{\prime }$	1.000	2.043	5.261	2.217	12.130
${\text{S}}_{01}^{\prime }$	1.000	2.000	2.000	0.750	5.000
${\text{S}}_{02}^{\prime }$	1.000	0.667	1.167	0.583	2.667

According to Eqs. 14 and 15, the correlation degree *R* and the correlation coefficient *ζ* of the first-level standard are shown in Table 6, and the correlation degree and correlation coefficient of the second-level standard are shown in Table 7.

**Table 6:** Correlation with the first-level air standard.

Correlation coefficient and correlation degree	*ζ*_1_	*ζ*_2_	*ζ*_3_	*ζ*_4_	*ζ*_5_	*R*_1_
$S_{1}^{\prime }$	1.000	0.807	0.333	0.637	0.591	0.674
$S_{2}^{\prime }$	1.000	0.638	0.333	0.558	0.955	0.697
$S_{3}^{\prime }$	1.000	0.988	0.522	0.708	0.333	0.710

**Table 7:** Correlation with the second-level air standard.

Correlation coefficient and correlation degree	*ζ*_1_	*ζ*_2_	*ζ*_3_	*ζ*_4_	*ζ*_5_	*R*_2_
$S_{1}^{\prime }$	1.000	0.832	0.333	0.646	0.431	0.648
$S_{2}^{\prime }$	1.000	0.716	0.333	0.591	0.405	0.609
$S_{3}^{\prime }$	1.000	0.775	0.536	0.743	0.333	0.677

According to Tables 6 and 7, all three classifications have the highest correlation with the first-level air standard. Therefore, the air quality in Ningxia Province in 2018 was associated with the first-level standard. More importantly, this result quantitatively indicates a correlation between the three classifications and the first-level air standard. The correlation degrees of the first classification, second classification and third classification with the first-level air standard are 0.674, 0.697 and 0.71, respectively. Therefore, it is clear that the gaps between the three classifications and the compared air standard are 0.326, 0.303 and 0.29. Moreover, the reason why the correlation degree cannot reach 1 is that some pollutant concentrations in the monitored data for these classifications are lower than the first-level air standard, and the remaining pollutant values are higher. Therefore, there is still room to continue to improve the air quality in the region. The region should continue to reduce the concentrations of pollutants and further improve the correlation degrees of all classifications of air with the first-level air standards.

## Conclusions

1. A new method of air quality assessment, *IGCFCCA*, is proposed. The advantage of the method is that it can quantitatively characterize the correlation degree between the current air quality and the corresponding standard level. Specifically, the results of this method indicated that the air quality in Ningxia Province in 2018 was correlated with first-level air in China’s air quality standard. The correlation degrees of the first classification, second classification and third classification of air quality with the first-level air standard are 0.674, 0.697 and 0.71, respectively. Therefore, the region should continue to reduce the concentrations of pollutants, especially PM_10_, PM_2.5_ and O_3_, and further improve the correlation degrees of all classifications with the first-level air standards. Notably, this method can be used in other industries.
2. The air quality in Ningxia Province in 2018 was classified into three classifications by ideal grey close function cluster analysis. Specifically, the relatively poor air quality in March, April and May and the comparatively better air quality in August and September correspond to the third classification, and the air quality in the remaining months corresponds to the second classification. In addition, the classification method can be used as a reference when establishing other classification standards, such as national standards, regional standards, and industry standards.

## Supplementary Information

Supplementary Information.
