# Supplementary material for: A new method for evaluating air quality using an ideal grey close function cluster correlation analysis method
Source: Sci Rep. 2021 Dec 2;11:23342. doi: 10.1038/s41598-021-02880-1 (PMC8639721; doi:10.1038/s41598-021-02880-1)
Supplement: Supplementary file 1 — Supplementary Information. [file 41598_2021_2880_MOESM1_ESM.doc]

**A New Method for Evaluating Air Quality Using an Ideal Grey Close Function Cluster Correlation Analysis Method**

**Xiaoling Ren^1*^, Zhenfu Luo^2^, Shuyu Qin^3^, Xinqian Shu^1*^, Yuanyuan Zhang^3^**

**The brief introduction of the other evaluation methods mentioned in the preface of the paper:**

(1) Air pollution index (API) method

Air Pollution Index (API - Abbreviation of Air Pollution Index) is a method to reflect and evaluate air quality. It simplifies the pollutant concentration that is not easy to understand into a single conceptual value, which is convenient to intuitively characterize the air quality and air pollution degree. The Air Pollution Index (API) classification criteria are as follows:

Level 1, API less than 50, excellent air quality.

Level 2, API 51~100, good air quality.

Level 3, API 101~150, minor pollution; API 151~200, mild pollution.

Level 4, API 201~300, moderate pollution.

Level 5, API > 300, heavy pollution.

(2) Ambient air quality index (AQI) method

AQI is a quantitative evaluation method. The process of AQI calculation and evaluation can be roughly divided into three steps: The first step is to calculate the air quality sub-index (IAQI for short) based on the measured concentration values of PM_2.5_, PM_10_, SO_2_, NO_2_, O_3_, CO and other pollutants by referring to the fractional concentration limits of each pollutant. The second step is to select the maximum value from the IAQI value of each pollutant and determine it as AQI. When AQI is greater than 50, the pollutant with the largest IAQI is determined as the primary pollutant. The third step is to determine the air quality level and category by referring to the AQI classification standard.

(3) Single factor index method

The single factor pollution index method is used to compare the measured concentration of a certain pollutant with the evaluation standard of this pollutant, and then determine the atmospheric category. In other words, each atmospheric monitoring parameter is compared with its evaluation standard, and the worst single index is finally selected to determine the atmospheric category, while weakening the role of other factors. Main pollution factors in the atmosphere can be determined by single factor pollution index evaluation.

(4) Green air pollution comprehensive index method

Green proposed to take SO_2_ and smoke coefficient (COH) as evaluation parameters. The method uses the daily average values of hope, warning and limit levels as hypothesis standards for SO_2_ and smoke coefficient. It expresses two pollution indexes SO_2_ and smoke coefficient in the form of power function. It stipulates that when SO_2_ or smoke coefficient reaches the hope, warning and limit levels, the pollution readings are 25, 50 and 100. Green averaged the SO_2_ and COH pollution indices to produce a composite index of air pollution.

(5) Aanalytic hierarchy process

The analytic hierarchy process is to decompose the evaluation object into different hierarchical units, and then calculate the weight of each layer by judging the feature vector of the matrix, and the largest weight is the main pollution factor.

1. Artificial neural network models

A neural network is an operational model consisting of a large number of nodes, or neurons, connected to each other. Each node represents a specific output function called activation function. Each connection between two nodes represents a weighted value of signals passing through the connection. This value is called the weight, which is equivalent to the memory of an artificial neural network. The output of the network varies according to the connection mode, weight value and excitation function of the network. Neural networks are usually approximations of some algorithm or function in nature.
